# Discovery, characterization and *in vivo* activity of pyocin SD2, a protein antibiotic from *Pseudomonas aeruginosa*

**DOI:** 10.1042/BCJ20160470

**Published:** 2016-07-28

**Authors:** Laura C. McCaughey, Inokentijs Josts, Rhys Grinter, Paul White, Olwyn Byron, Nicholas P. Tucker, Jacqueline M. Matthews, Colin Kleanthous, Cynthia B. Whitchurch, Daniel Walker

**Affiliations:** * The Ithree Institute, University of Technology Sydney, Ultimo, New South Wales 2007, Australia; † Department of Biochemistry, University of Oxford, South Parks Road, Oxford, OX1 3QU, U.K.; ‡ Institute of Infection, Immunity and Inflammation, College of Medical, Veterinary and Life Sciences, University of Glasgow, Glasgow, G12 8QQ, U.K.; § School of Life Sciences, College of Medical, Veterinary and Life Sciences, University of Glasgow, Glasgow, G12 8QQ, U.K.; ∥ Strathclyde Institute for Pharmaceutical and Biomedical Sciences, University of Strathclyde, Glasgow, G4 0RE, U.K.; ¶ School of Molecular Bioscience, University of Sydney, New South Wales 2008, Australia

**Keywords:** antibiotics, bacteriocins, common polysaccharide antigen, outer membrane, *Pseudomonas aeruginosa*, pyocins

## Abstract

Increasing rates of antibiotic resistance among Gram-negative pathogens such as *Pseudomonas aeruginosa* means alternative approaches to antibiotic development are urgently required. Pyocins, produced by *P. aeruginosa* for intraspecies competition, are highly potent protein antibiotics known to actively translocate across the outer membrane of *P. aeruginosa.* Understanding and exploiting the mechanisms by which pyocins target, penetrate and kill *P. aeruginosa* is a promising approach to antibiotic development. In this work we show the therapeutic potential of a newly identified tRNase pyocin, pyocin SD2, by demonstrating its activity *in vivo* in a murine model of *P. aeruginosa* lung infection. In addition, we propose a mechanism of cell targeting and translocation for pyocin SD2 across the *P. aeruginosa* outer membrane. Pyocin SD2 is concentrated at the cell surface, via binding to the common polysaccharide antigen (CPA) of *P. aeruginosa* lipopolysaccharide (LPS), from where it can efficiently locate its outer membrane receptor FpvAI. This strategy of utilizing both the CPA and a protein receptor for cell targeting is common among pyocins as we show that pyocins S2, S5 and SD3 also bind to the CPA. Additional data indicate a key role for an unstructured N-terminal region of pyocin SD2 in the subsequent translocation of the pyocin into the cell. These results greatly improve our understanding of how pyocins target and translocate across the outer membrane of *P. aeruginosa.* This knowledge could be useful for the development of novel anti-pseudomonal therapeutics and will also support the development of pyocin SD2 as a therapeutic in its own right.

## Abstract (precis)

Here we propose a mechanism of cell targeting and translocation for an S-type pyocin, pyocin SD2. Understanding and exploiting the mechanisms by which pyocins target, penetrate and kill *Pseudomonas aeruginosa* is a promising approach to antibiotic development.

## INTRODUCTION

*Pseudomonas aeruginosa* is a Gram-negative opportunistic pathogen notable for being the leading cause of mortality in patients with cystic fibrosis [1]. Additionally, *P. aeruginosa* is the principle organism associated with burn wound infections [2], the most prevalent Gram-negative bacteria associated with nosocomial and ventilator associated pneumonia [3] and the second most common cause of catheter-associated urinary tract infections [4]. Together with many bacterial pathogens, there is an increasing prevalence of multi-drug resistance (MDR) in *P. aeruginosa*, which has resulted in limited treatment options for patients [5]. In 2013 there were an estimated 51000 health-care associated *P. aeruginosa* infections in the U.S.A., more than 13% of which were classed as MDR [6]. Furthermore, the prevalence of pan-drug resistant *P. aeruginosa* isolates (non-susceptibility to all antibiotics in all antimicrobial classes) is also increasing, resulting in a return to a pre-antibiotic era for many patients with *P. aeruginosa* infections [7–12]. Therefore, there is an urgent requirement to develop novel antibiotics to treat this drug resistant pathogen.

*P. aeruginosa* utilizes intrinsic, acquired and adaptive mechanisms for antibiotic resistance. These include but are not limited to: inherently low levels of outer membrane permeability; expression of multi-drug efflux pumps and antibiotic degrading enzymes (e.g. β-lactamases); accumulation of mutations in antibiotic targets; acquisition of genetic elements encoding resistance genes; alteration of growth state (e.g. biofilm formation) and phenotypic variation (e.g. appearance of small colony and mucoid variants in the population). The outer membrane of *P. aeruginosa* is estimated to be 10–100-fold less permeable to small hydrophilic molecules, including antibiotics, than that of *Escherichia coli* [13]. As well as this intrinsic outer membrane impermeability, *P. aeruginosa* employs several mechanisms of altering the outer membrane composition to reduce both the lipid-mediated uptake of hydrophobic antibiotics and porin-mediated diffusion of hydrophilic antibiotics across the outer membrane. These include the loss of the carbapenem-specific porin OprD, reduction in cell envelope Mg^2+^ and Ca^2+^, alterations in lipopolysaccharide (LPS) O-antigen composition and alterations of the lipid A and core oligosaccharide moieties of LPS [5,14,15]. As the outer membrane of *P. aeruginosa* poses a formidable barrier to antibiotic treatment, identifying and exploiting new ways to target and penetrate the outer membrane is essential for the successful development of new anti-pseudomonal antibiotics.

Pyocins are protein antibiotics produced by *P. aeruginosa* for intraspecies competition [16]. They can take the form of a DNase, tRNase, pore former or they can inhibit peptidoglycan synthesis, and they parasitize *P. aeruginosa*-specific nutrient uptake pathways to achieve active transport across the outer membrane. These extremely potent anti-pseudomonal proteins represent excellent tools for antibiotic development; not only can they provide great insight into new ways to target and penetrate the outer membrane they are also promising therapeutics in their own right. To date the outer membrane receptors for three pyocins have been identified. These are FpvAI, FpvAII and FptA, all of which are involved in the uptake of iron-siderophore complexes [17–20]. Pyocin L1 has also been shown to bind to the common polysaccharide antigen (CPA) of *P. aeruginosa* LPS, a homopolymer of the rare deoxyhexose D-rhamnose, as a way of targeting cells [21]. Outside the identification of outer membrane receptors utilized by pyocins, little is known about the mechanisms involved in pyocin translocation and much of our understanding, including the assignment of domain functionalities (e.g. receptor binding, translocation and cytotoxic domains [16]) is inferred from research on colicins (protein antibiotics produced by and active against *E. coli* [22]).

In the case of the E-type colicins, two outer membrane proteins are required for binding and translocation across the outer membrane: a high-affinity receptor and a translocator. Once bound to their high-affinity receptors, via their receptor-binding domains, colicins search the outer membrane landscape for their translocators via lateral diffusion. This is facilitated by the 45° orientation of the elongated receptor-binding domain of the colicin to the membrane plane, known as the ‘fishing pole’ mechanism [23]. Translocation then requires the N-terminal intrinsically unstructured translocation domain of the colicin to thread through the central pore of the translocator protein where it can interact with components of the import machinery (the TonB system or the Tol system) via specific Tol or TonB boxes [23–25]. The next steps in the translocation of E-type colicins across the outer membrane remain largely unknown. However, the end goal of cell death is known to occur via a DNase, tRNAse, rRNase or pore forming functionality. For the smaller, globular colicin M, which uses only one copy of FhuA for binding and translocation, the intrinsically unstructured translocation domain is much shorter (37 residues) but is similarly positioned at the N-terminus and includes a TonB box (residues 2–8) [26,27]. Colicin N is unique among colicins in that it binds to both LPS on the surface of *E. coli* and to the general porin OmpF in order to cross the outer membrane [28]. It is unlikely that one model can describe the mechanism of binding and translocation for both pyocins and colicins as these form a diverse group of proteins, differing in size, structure and activity [16,21,22,29,30].

In this work we have identified and purified three pyocins, SD1, SD2 and SD3, with N-terminal domains homologous with pyocins S1, S2 and S3 respectively, and C-terminal cytotoxic domains that are homologous with the tRNase domain of colicin D. Through *in vivo* studies we show that pyocin SD2 can afford protection against a lethal *P. aeruginosa* lung infection in a murine model, demonstrating its potential for therapeutic development. *In vitro* characterization of pyocin SD2 indicates that only one copy of the outer membrane receptor FpvAI is used by pyocin SD2 and the TonB box on FpvAI is essential for pyocin SD2 activity. Furthermore, the first 17 amino acids of pyocin SD2 are essential for cytotoxic activity, but are not involved in receptor binding. In addition, we show that pyocin SD2 binds to the CPA of *P. aeruginosa* LPS and that CPA-binding is required for high efficiency killing of *P. aeruginosa*. Together these results suggest that pyocin SD2 utilizes both the CPA and FpvAI to target *P. aeruginosa* and that a predicted unstructured N-terminal region of pyocin SD2 is critical for subsequent translocation.

## MATERIALS AND METHODS

### Strains and plasmids

The strains and plasmids used in this study are described in Supplementary Table S1. Strains of *E. coli* and *P. aeruginosa* were grown in LB broth at 37°C.

### Cloning of pyocins

Pyocin S2 was cloned as previously described [31]. Pyocin SD2-imSD2 was amplified from the genomic DNA of the producing strain *P. aeruginosa* MSH10 by PCR using primers designed to introduce an NdeI site at the start of the *pyoSD2* gene (TGT CAA CAT ATG GCT GTC AAT GAT TAC GAA CC) and an XhoI site at the end of the *pyoSD2-im* gene (TGT CAA CTC GAG TAT GTA TTT ATA TTC TTT CAA TAG ATC ACT C). For pyocin S2Δ17 primers were designed to introduce an NdeI site 17 amino acids into the *pyoS2* gene (TGT CAA CAT ATG GGT GGT GGG CGT GAC ATA AT). For pyocin S2Δ318, primers were designed to introduce an NdeI site before residue 318 of the *pyoS2* gene (C GCT CGT CAA GCG GCG CAT ATG GCT GCC AAT ACT TAT G). The N-terminal portions were removed by digestion with NdeI. For pyocin S2(1–209), primers were designed to introduce an XhoI site after residue 209 of the *pyoS2* gene by site-directed mutagenesis (G GAG GCG GAC TAC AAG CTC GAG AAG GCA AAT GTC GAG). The PCR product was digested with XhoI to remove intervening DNA. The PCR products were ligated into the corresponding sites of the *E. coli* expression vector pET21a to give pETPyoSD2 which encodes pyocin SD2-imSD2, pETPyoS2Δ17 which encodes pyocin S2Δ17-imS2, pETPyo209 which encodes pyocin S2(209) and pETPyoS2Δ318 which encodes pyocin S2Δ318-imS2, all with C-terminal His_6_ tags. The gene encoding pyocin S5 was similarly amplified from the genomic DNA of strain PAO1 using primers designed to introduce an NdeI site at the start of the gene (GAG ACA TAT GTC CAA TGA CAA CGA AGT AC) and an XhoI site after the stop codon (TTT GAC GTC TCG AGT TAA ATG GAT ATT ACA AGA TTG TTT GC). The digested PCR product was ligated into pET15b to give pETPyoS5, which encodes pyocin S5 with an N-terminal His_6_-tag. The genes encoding pyocin AP41 and its immunity protein (ImAP41) were amplified from the genomic DNA of *P. aeruginosa* C763 by PCR using primers designed to introduce an NdeI site at the start of the pyocin encoding gene (ACA GAT CAT ATG AGC GAC GTT TTT GAC CTT GG) and an XhoI in place of the stop codon of the ImAP41 encoding gene (ACA GAT CTC GAG GCC AGC CTT GAA GCC AGG G). The PCR product was digested with NdeI and XhoI and ligated into the corresponding sites of the *E. coli* expression vector pET21a to give pETPyoAP41, which was used for the production of the pyocin AP41–ImAP41 complex in which ImAP41 carries a C-terminal His_6_-tag. Pyocins SD1, SD3 and SD2Δ216 were synthesized by GenScript, US, and ligated into pET21a using NdeI and XhoI restriction sites to produce complexes in which the immunity proteins carry C-terminal His_6_-tags.

### Purification of pyocins

All pyocins and pyocin variants used in this study were overexpressed from *E. coli* BL21(DE3)pLysS carrying the corresponding plasmids. For each pyocin, 5 litres of LB broth containing 100 μg·ml^−1^ of ampicillin was inoculated (1:100) from an overnight culture and cells were grown at 37°C in a shaking incubator to an *OD*_600_=0.6. Protein production was induced by the addition of 1.0 mM isopropyl β-D-1-thiogalactopyranoside (IPTG) and cells were grown at 37°C for a further 3.5 h before harvesting by centrifugation. Cells were resuspended in 20 mM Tris/HCl, 500 mM NaCl, 20 mM imidazole, pH 7.5, lysed and the cell debris separated by centrifugation. The cell-free lysate was applied to a 5 ml HisTrap column (GE Healthcare) equilibrated in 20 mM Tris/HCl, 500 mM NaCl, 20 mM imidazole, pH 7.5, and the pyocins eluted over a 20–500 mM imidazole gradient. Pyocin containing fractions were pooled, dialysed overnight into 50 mM Tris/HCl, 200 mM NaCl, pH 7.5 and remaining contaminants removed by gel filtration chromatography on a Superdex S200 16/60 column or Superdex S75 26/60 column (GE Healthcare) equilibrated in the same buffer. The proteins were concentrated using a centrifugal concentrator (Amicon Ultra 15, MerckMillipore) with a molecular weight cut off of 5 kDa and stored at −80°C. Prior to use protein concentrations were determined using a NanoDrop (NanoDrop ND1000, Thermo Scientific).

### Overlay spot plate method

One hundred and fifty microlitres of test strain culture at *OD*_600_=0.6 was added to 6 ml of 0.8% agar and poured over an LB agar plate. Five or ten microlitres of purified pyocin at various concentrations was spotted on to overlay plates and incubated for 24 h at 37°C. Clear zones indicate killing.

### Bactericidal activity assay in liquid culture

For pyocin SD2 receptor saturation experiments, *P. aeruginosa* PAO1 was grown in LB broth with shaking at 37°C to *OD*_600_=0.2. The culture was then divided into four separate flasks. Pyocin SD2 was added to a final concentration of 6.3 μg·ml^−1^, 63 μg·ml^−1^ and 630 μg·ml^−1^ to three separate flasks. Growth was continued with shaking at 37°C and the *OD*_600_ of the untreated and pyocin-treated cultures were monitored at 20 min intervals. The highest concentration of pyocin SD2 used (630 μg·ml^−1^) resulted in 13.7×10^6^ pyocin SD2 molecules per cell.

For pyocin SD2-CPA killing efficiency experiments, cells at a middle-log phase were adjusted to an *OD*_600_=0.4. One millilitre aliquots of the cells were mixed with pyocin SD2 at a final concentration of 3 μM. The mixtures were incubated at 37°C with shaking at 180 rpm for 30 min, 60 min, 90 min and 180 min. The *OD*_600_ was recorded at each time point and the cell aliquots from each time point were used to calculate colony-forming units (CFU).

### Secondary structure prediction and sequence alignments

To predict secondary structure features PSIPRED-software was used [32]. The prediction was applied to the whole sequence. Pyocin protein sequence alignments were created using CLC Main Workbench 7. The alignments were manually checked for conserved regions and domain boundaries. Precise delineation of the FpvAI binding domain will be reported elsewhere.

### SAXS

SAXS was carried out on the X33 beamline at the Deutsches Elektronen Synchrotron (DESY). Data were collected on samples of pyocin in the range of 0.5–5 mg·ml^−1^. Buffer was scattered before and after each sample and an average of the buffer scattering was subtracted from the sample scattering. The data obtained for each sample were analysed using PRIMUS [33], merging scattering data at low angles with high angle data. The pair distance distribution function, *p*(*r*), was obtained by indirect Fourier transform of the scattering intensity using GNOM [34]. A Guinier plot [ln(I) vs *S*^2^] was used to calculate the radius of gyration, *R*_g_, of the pyocin complex. An *ab initio* model of the protein complex in solution was built using DAMMIF [35]. Twenty independent reconstructions produced similar shapes and these were averaged using DAMAVER [36].

### LPS purification and isolation of LPS-derived polysaccharide

LPS was purified from 1 litre cultures of *P. aeruginosa* strains as described previously, with modifications including the omission of the final trifluoroacetic acid hydrolysis and chromatography steps [37]. Cells were grown for 20 h at 37°C, pelleted by centrifugation at 6000 ***g*** for 20 min and resuspended in 50 mM Tris/HCl, pH 7.5 containing lysozyme (2 mg·ml^−1^) and DNase I (0.5 mg·ml^−1^). Cells were lysed by sonication and the cell lysate was incubated at 20°C for 30 min before EDTA was added to a final concentration of 2 mM. An equal volume of aqueous phenol was added and the solution was heated at 70°C for 20 min, with vigorous mixing. The solution was then cooled on ice for 30 min, centrifuged at 7000 ***g*** for 20 min and the aqueous phase extracted. Proteinase K was added to a final concentration of 0.05 mg·ml^−1^ and dialysed for 12 h against 2×5 litres dH_2_O. LPS was pelleted by ultracentrifugation at 100000 ***g*** for 1 h, resuspended in dH_2_O and heated to 60°C for 30 min to remove residual proteinase K activity. LPS-derived carbohydrates were isolated by heating LPS in 2% acetic acid for 1.5 h at 96°C. Lipid A was removed by centrifugation at 13500 ***g*** for 3 min followed by extraction with an equal volume of chloroform. The aqueous phase was then lyophilized.

### Isothermal titration calorimetry

Isothermal titration calorimetry (ITC) experiments were performed on a VP-ITC microcalorimeter or using a MicroCal iTC200 microcalorimeter at 25°C (MicroCal). Pyocins or pyocin variants were used as the titrant at various concentrations (Table 2, Supplementary Table S2) with LPS-derived carbohydrates dissolved at 1 mg·ml^−1^ or 3 mg·ml^−1^ (Table 2, Supplementary Table S2) in the chamber. Prior to analysis protein concentrations were determined using a NanoDrop (NanoDrop ND1000, Thermo Scientific). Reactions were performed in 0.2 M sodium phosphate buffer, pH 7.5. For curve fitting the molar concentration of LPS-derived CPA containing carbohydrate chains was estimated at 20 μM, based on an estimated average molecular weight of 10 kDa for CPA containing polysaccharides and estimating the percentage of total LPS represented by CPA containing carbohydrates as 20% of the total by weight [38]. The *N*-value equals the stoichiometry when the concentrations used for fitting are correct and 100% active. As the concentration of CPA in the cleaved LPS sugars is an estimate the stoichiometry implied by the fit is likely to be unreliable. However, the use of this estimated value has no impact on the reported parameters of Δ*H*, Δ*S* and *K*_d_. All samples were degassed extensively prior to the experiments. The heats of dilution for protein into buffer for each titration were obtained and subtracted from the raw data. Data were fitted to a single-binding site model with Microcal LLC Origin software. Errors reported in the text are the S.E.M. from the average of two experiments. Errors reported in the figure legends are the standard error of the fit for the experiment shown in the figure. All ITC binding parameters and errors are reported in Table 2. For pyocin SD2Δ216 to PAO1 LPS sugars a flat curve was obtained due to the *K*_d_ being in the mid micromolar range. As these curves lacked an inflection point the *N* value was fixed to a constant value during the fit. This value was 0.1, which was the approximate *N*-value for pyocins SD2, S2, S5 and SD3 binding to PAO1 LPS sugars.

### Far-UV CD

Proteins in 0.2 M sodium phosphate buffer, pH 7.5 at 25°C were diluted to a concentration of 15–150 μg·ml^−1^ and far-UV CD spectra were recorded on a J-815 spectropolarimeter (Jasco) in a 0.1 cm quartz cuvette using scanning speed of 20 nm·min^−1^, a step size of 0.5 nm and digital integration time of 1 s. Spectra are an average of three scans and are buffer baseline corrected. The final spectra were represented by molar ellipticity, Δ*ε* (mdeg·M^−1^·cm^−1^).

### Ethics statement

All animal experiments were performed in accordance with the UK Animals (Scientific procedures) Act, authorized under a UK Home Office License, and approved by the animal project review committee of the University of Glasgow. Animal studies were not randomized and blinding was not possible in this study. The project license number assigned by the animal project review committee of the University of Glasgow was 60/4361.

### Murine model of acute *P. aeruginosa* lung infection

Six-week-old, female, murine pathogen free C57/BL6 mice (Charles Rivers Laboratories) weighing 15–21 g were inoculated intranasally with 25 μl of bacterial culture containing approximately 10^7^ CFU of *P. aeruginosa* PAO1 after induction of anaesthesia with isofluorane. Pyocins SD2 and S2 dissolved in PBS (25 μl at 3 mg·ml^−1^) (*n*=6) were delivered via the intranasal route, after induction of anaesthesia with isofluorane, 1 h post-infection and were administered only once. To determine if mice could survive infection after pyocin SD2 treatment, mice were monitored closely, culled by carbon dioxide asphyxiation when required as determined by a scoring system or culled at the pre-determined 24 h time point. The scoring system assessed illness (mild/moderate/severe) based on whether the mice were starey, hunched, lethargic or moribund. The maximum overall score that resulted in a mouse being culled was seven (two for starey, hunched and lethargic and one for moribund). However, if a mouse scored one for moribund but did not score six in the other categories, it was still culled. For CFU determination, lungs were removed aseptically and kept on ice in 750 μl of PBS until homogenized. Serial 10-fold dilutions of the homogenized lung were plated on *Pseudomonas* selective agar (20 g peptone, 1.5 g K_2_HPO_4_, 1.5 g MgSO_4_·7H_2_O, 10 ml glycerol, 15 g agar, 0.025 g Irgasan per litre) and incubated at 37°C for 24 h and then room temperature for 24 h before the colonies were counted. To determine pyocin SD2 resistance of recovered colonies the overlay spot plate method was used.

## RESULTS AND DISCUSSION

### Discovery and characterization of pyocins SD1, SD2 and SD3

**Figure 1:**
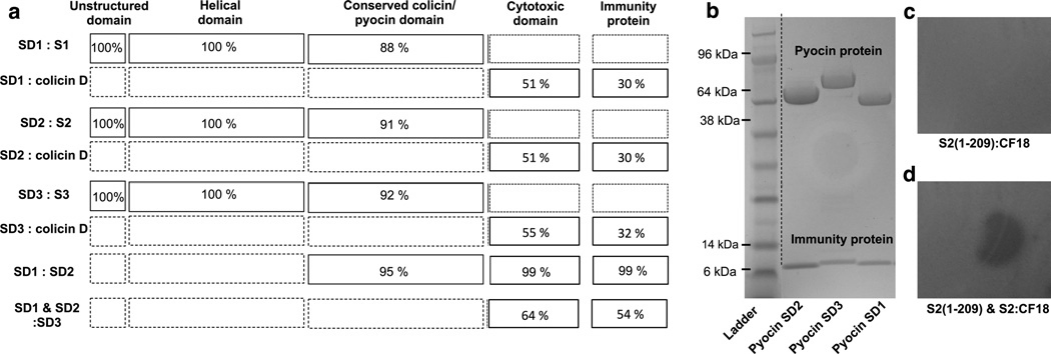
Discovery and purification of pyocins SD1, SD2 and SD3 (**a**) Comparison of the amino acid % identity for pyocins SD1, SD2 and SD3, with pyocins S1, S2, S3 and colicin D. The cytotoxic domains of pyocins SD1, SD2 and SD3 are homologous with colicin D and the N-terminal domains are homologous with the respective pyocins. Pyocins SD1 and SD2 are nearly identical in their cytotoxic domains and immunity proteins. (**b**) SDS/PAGE gel (4–12%) of purified pyocins SD1 (63 kDa), SD2 (72 kDa) and SD3 (79 kDa) and their immunity proteins (10 kDa) post purification. (**c**) Pyocin S2(1–209) (1 mg·ml^−1^) is not active against CF18. (**d**) Inhibition of pyocin S2 killing (1 μg·ml^−1^) by pyocin S2(1–209) (1 mg·ml^−1^).

The genomes of 15 *P. aeruginosa* clinical and environmental isolates [39], including PAO1 [40], PA14, PA7 and LESB58, were analysed for putative pyocin producing genes by searching for sequences homologous with those of known bacteriocin genes. We identified three putative pyocin and immunity protein gene pairs. These encoded tRNase-type pyocins and their associated immunity proteins [39] (Table 1). The cytotoxic domains of these putative pyocins share between 51% and 55% amino acid identity with the cytotoxic domain of colicin D (Figure 1). Colicin D is a tRNase-type colicin [41] and four of the six residues critical for cytotoxic activity in colicin D (K608, H611, S677 and W679) are conserved in the putative pyocins. The immunity protein genes share between 30% and 32% amino acid identity with the colicin D immunity protein (Figure 1). Sequences N-terminals to the cytotoxic domains of these putative pyocins share significant homology with pyocins S1, S2 or S3. Consequently, we designated these putative pyocins as pyocin SD1, pyocin SD2 and pyocin SD3 respectively. To determine the killing spectrum of pyocins SD1, SD2 and SD3 we cloned the pyocin and immunity protein open reading frames for each pyocin-immunity protein pair into the pET21a vector, resulting in a His_6_-tag on the C-terminal end of the immunity protein. Pyocins SD1-imSD1, SD2-imSD2 and SD3-imSD3 were expressed in BL21(DE3)pLysS cells and purified by nickel affinity and size exclusion chromatography. Pyocins SD1, SD2 and SD3 contain 591, 662 and 740 amino acids respectively, in the pyocin protein and range in size from 63 kDa to 79 kDa. The immunity proteins of all three contain 90 amino acids and are 10 kDa in size (Table 1, Figure 1b). Pyocin SD1 killed 5/63 clinical and environmental *P. aeruginosa* isolates tested, pyocin SD2 killed 6/63 isolates including PAO1, and pyocin SD3 showed activity against 15/63 isolates. Killing was observed down to a concentration of approximately 12 nM for all three pyocins. A recent review identified genes encoding pyocins SD2 and SD3 using bioinformatics approaches and designated them as pyocins S11 and S12 [42]. However, as pyocins S7–S10 have not been experimentally validated, to maintain consistency in the literature, we propose the alternative designations SD2 and SD3.

**Table 1:** Genome locations and sizes of pyocins SD1, SD2 and SD3 *Pyocin protein also present in 2 other *p. aeruginosa* strains on NCBI. Immunity protein present in 40 other *P. aeruginosa* strains on NCBI. ^†^Pyocin protein also present in 102 other *p. aeruginosa* strains on NCBI. Immunity protein present in 40 other *P. aeruginosa* strains on NCBI. ^‡^Pyocin protein also present in 2 other *P. aeruginosa* strains on NCBI. Immunity protein present in 17 other *P. aeruginosa* strains on NCBI. 100% coverage and > 90% identity used for NCBI search.

	Strain identified in	Pyocin and immunity protein gene position	Pyocin (amino acids)	Immunity protein (amino acids)	Pyocin+immunity protein (kDa)
Pyocin SD1*	PA62	4170921–4172988 (Not annotated)	591	90	63.4+10.1
Pyocin SD2^†^	MSH10	4109138–4111400	662	90	71.5+10.1
		(MSH10-3855 and MSH10-3856)			
Pyocin SD3^‡^	PA7	3134080–3136575	740	90	78.5+10.3
		(PA7-3036 and PA7-3037)			

### Domain organization

**Figure 2:**
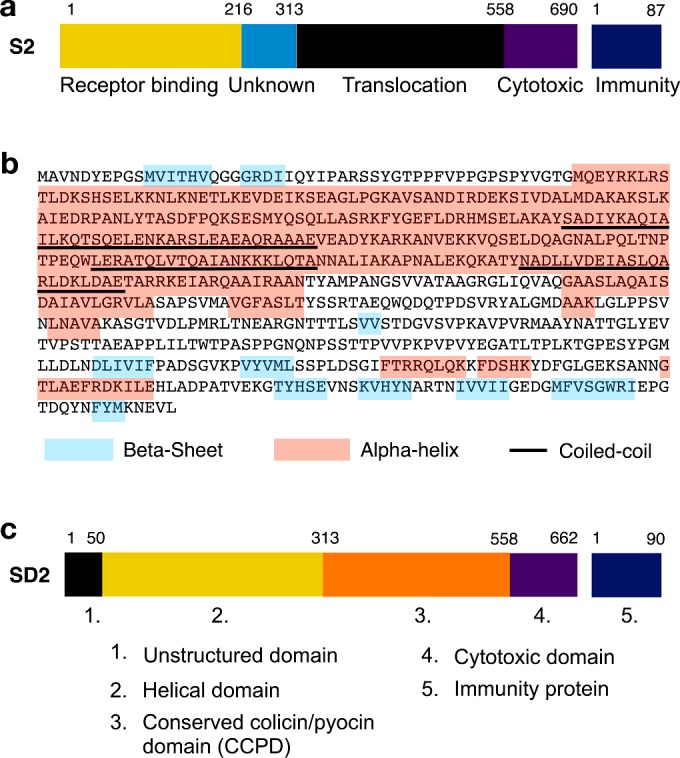
Sequence alignment and secondary structure predictions for pyocin SD2 (**a**) Previously proposed domain architecture of pyocin S2. (**b**) Predicted secondary structure features of pyocin SD2 using PSIPRED-software [32]. The first 50 residues of pyocin SD2 have little regular secondary structure and are rich in proline and glycine residues. (**c**) Newly proposed domain architecture of pyocin SD2.

The domain organization of colicin-like pyocins such as S2 and SD2 is thought to differ from that of the colicins, which have an N-terminal translocation domain, followed by a central receptor-binding domain and a C-terminal cytotoxic domain, in that the order of the translocation and receptor-binding domains is reversed [16,22]. In addition, some colicin-like pyocins appear to possess an additional domain of unknown function [16,22]. Therefore, for pyocins S2/SD2 the domain order from the N-terminus is currently thought to be: receptor-binding domain, unknown domain (not present in all pyocins), translocation domain and cytotoxic domain (Figure 2a). Reversal of the order of the receptor binding and translocation domains in pyocins and colicins has implications in terms of pyocin translocation. A centrally positioned translocation domain for pyocins does not logically allow for an extended unstructured region to pass across the outer membrane to the periplasm, as has been demonstrated for colicins [23]. For this reason the domain architecture of pyocins S2/SD2 was reassessed using secondary structure predictions and sequence alignments of multiple pyocins. The first 50 amino acids of pyocins S2/SD2 are predicted to largely lack regular secondary structure and are rich in proline and glycine (Figure 2b). Amino acids 50–318 of pyocins S2/SD2 are predicted to be helical with a coiled-coil structure and do not appear to be two separate domains as previously annotated (Figure 2b). The central domain of pyocins was previously designated the ‘translocation domain’ due to homology with the globular domain at the N-terminus of many colicins [16]. However, it has yet to be experimentally demonstrated that this conserved colicin/pyocin domain (CCPD) has a direct role in colicin/pyocin translocation. In fact, the role of this domain in the mode of action of these proteins remains a mystery [23,25]. Sequence alignments of pyocins S2/SD2 indicate that the cytotoxic domain of pyocin SD2 encompasses the C-terminal residues 558–662. Based on these structural and sequence alignment predictions the domain architecture of pyocins S2/SD2 would be better described from the N-terminus as follows: unstructured domain, helical domain, conserved colicin/pyocin domain (CCPD) and cytotoxic domain (Figure 2c).

### Pyocin SD2 is highly elongated

**Figure 3:**
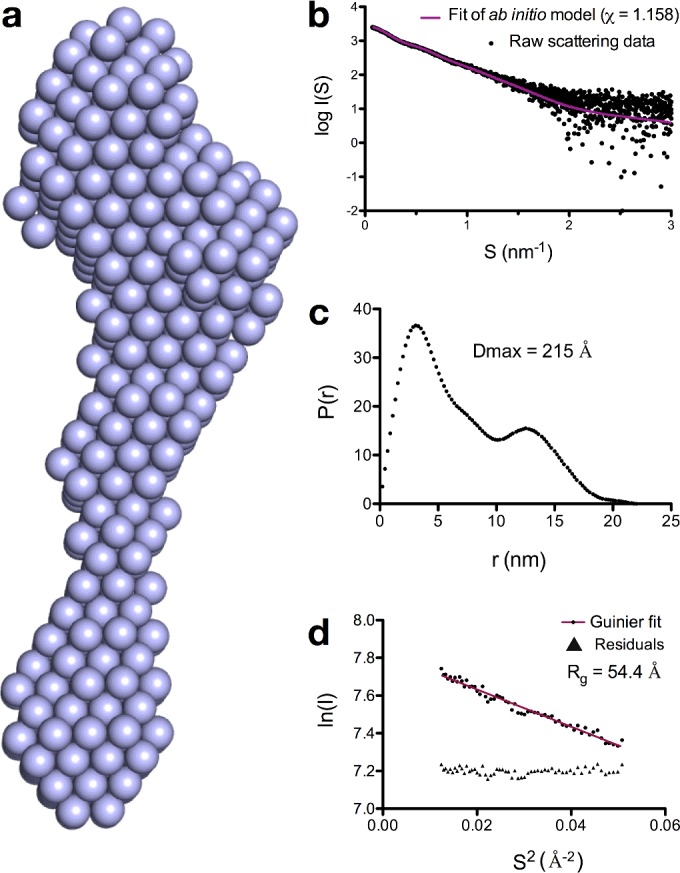
Small angle X-ray scattering model of pyocin SD2 (**a**) *Ab initio* model of pyocin SD2 computed with DAMMIF and averaged with DAMAVER. (**b**) Overlay of the experimentally determined pyocin SD2 SAXS curve (black points) with the fit of the *ab initio* model (purple line), produces a good fit (*χ*=1.158). (**c**) Pair-distance distribution plots from experimental scattering data for pyocin SD2 suggest that this protein is an elongated multi-domain protein in solution. *D*_max_=215 Å. (**d**) Guinier plot of scattering data indicates that pyocin SD2 is monomeric in solution. Radius of gyration is 54.4 Å.

To gain insight into the structural basis of pyocin translocation, we obtained small angle X-ray scattering (SAXS) data for pyocin SD2 in complex with its immunity protein, ImSD2. Data were obtained for a range of pyocin SD2 concentrations. An *ab initio* model of the pyocin SD2–imSD2 complex was generated using DAMMIF and was a good fit to the experimental scattering data (*χ*=1.158) (Figures 3a and 3b). *Ab initio* modelling of the pyocin SD2 envelope produced a dumbbell shape (Figure 3a), which correlates with the presence of two maxima on the pair-distance distribution plot for the pyocin SD2–imSD2 complex (Figure 3c). The maximum particle dimension obtained from this graph [*D*_max_=215 Å (1 Å=0.1 nm)] shows the pyocin SD2–imSD2 complex to be an elongated molecule (Figure 3c). Guinier analysis of the experimental scattering data (Figure 3d) shows that pyocin SD2–imSD2 has a radius of gyration of 54.4 Å and is monomeric in solution. These data show that similar to the E-type colicins, pyocin SD2 is highly elongated with a large globular region at one end, and a smaller globular region at the other end.

### Pyocins S2/SD2 require a single copy of FvpAI for receptor binding and translocation

**Figure 4:**
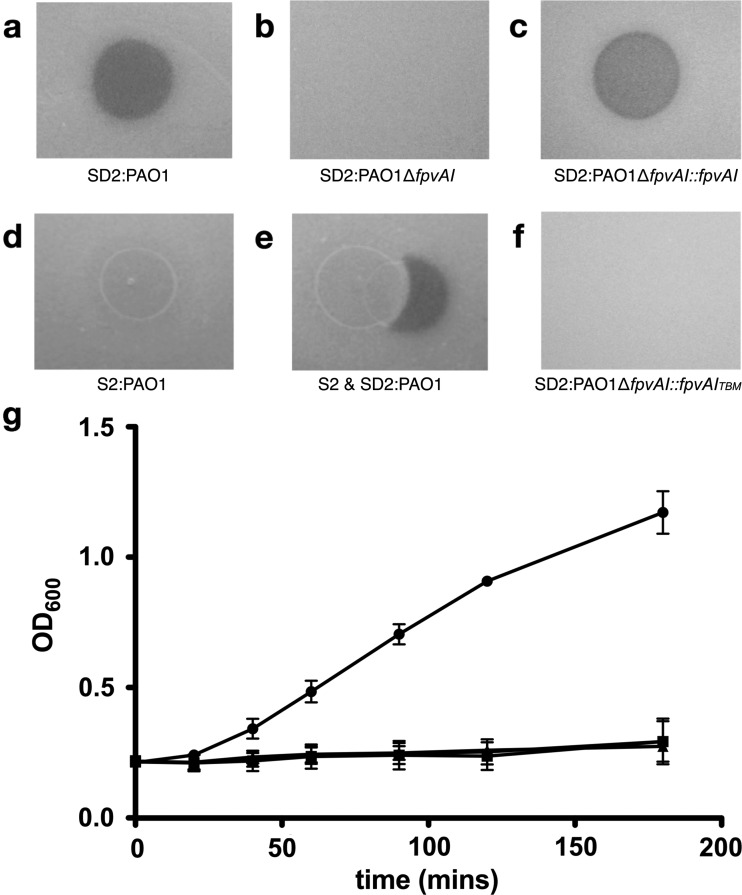
Pyocin SD2 utilizes the outer membrane protein FpvAI Ten microlitres of purified pyocin was spotted on to a growing lawn of cells. Clear zones indicate cell death. (**a**) Inhibition of growth of *P. aeruginosa* PAO1 by pyocin SD2 (1 mg·ml^−1^). (**b**) Pyocin SD2 (1 mg·ml^−1^) is not active against PAO1Δ*fpvAI*. (**c**) Pyocin SD2 killing (1 mg·ml^−1^) is restored against PAO1Δ*fpvAI*::*fpvAI*. (**d**) Pyocin S2 (2 mg·ml^−1^) is not active against PAO1. (**e**) Inhibition of pyocin SD2 killing (1 mg·ml^−1^) by pyocin S2 (2 mg·ml^−1^) against PAO1. (**f**) Pyocin SD2 (1 mg·ml^−1^) is not active against PAO1Δ*fpvAI*::*fpvAI*_TBM_. (**g**) *P. aeruginosa* PAO1 was grown in LB broth with shaking at 37°C to *OD*_600_=0.2. The culture was then divided into four separate flasks. Pyocin SD2 was added to a final concentration of 6.3 μg·ml^−1^ (square), 63 μg·ml^−1^ (triangle) and 630 μg·ml^−1^ (star) to three separate flasks. Growth was continued with shaking at 37°C and the *OD*_600_ of the untreated (circle) and pyocin-treated cultures were monitored at 20 min intervals. Bars represent mean ± S.E.M. (*n*=2). The highest concentration of pyocin SD2 used (630 μg·ml^−1^) resulted in 13.7×10^6^ pyocin SD2 molecules per cell. Killing of PAO1 by pyocin SD2 was seen at all three pyocin concentrations.

The very high level of sequence identity between the non-cytotoxic domains of pyocin S2 and pyocin SD2 suggests that both proteins utilize the same cell-surface receptor. For pyocin S2 this has previously been shown to be the ferripyoverdine receptor FpvAI [17]. To confirm FpvAI is also the receptor for pyocin SD2, its activity was assessed against PAO1Δ*fpvA*, a mutant of the pyocin SD2-sensitive strain PAO1 with the *fpvA* gene deleted [43]. Pyocin SD2 was not active against PAO1Δ*fpvA* (Figures 4a and 4b) and activity was restored after complementation with the *fpvAI* gene (Figure 4c). Consistent with this, pyocin SD2 killing of *P. aeruginosa* PAO1 could be inhibited by the addition of pyocin S2 in competition overlay spot plate assays (PAO1 is a pyocin S2 producing strain and so is not killed by pyocin S2 due to production of the cognate immunity protein ImS2) (Figures 4d and 4e). These data show that pyocin SD2 uses FpvAI as a cellular receptor.

In the case of the highly elongated colicin Ia, two copies of its receptor Cir are required for colicin activity, with one copy acting as a high-affinity receptor and the other as a translocation pore [44]. Killing of *E. coli* is inhibited at high colicin Ia concentrations as the high-affinity receptor-binding domain of colicin Ia binds all copies of Cir. This leaves no unbound Cir molecules to interact with the intrinsically unstructured translocation domains of the bound colicins [44]. To determine if pyocin SD2, which has a highly elongated structure similar to colicin Ia, requires two copies of FpvAI on the cell surface for receptor binding and translocation, the growth of *P. aeruginosa* PAO1 in the presence of 1×, 10× and 100× the MIC of pyocin SD2 was monitored (Figure 4g). All three concentrations of pyocin SD2 were able to inhibit the growth of *P. aeruginosa* PAO1, with no reduction in killing observed with increasing concentration. At the highest concentration of pyocin used there were 13.7×10^6^ pyocin SD2 molecules present per cell. Overlay spot plate assays were also used to test for FpvAI saturation by pyocin SD2. The lack of a central zone of bacterial growth or ‘halo’ within the clear zone of pyocin SD2 killing on the spot plates shows that there is no receptor saturation at high pyocin SD2 concentrations (300 μM). A lack of receptor saturation in the growth assay and the spot plate assay indicates that pyocin SD2 requires only one copy of FpvAI for cell entry.

These data suggest either pyocins S2/SD2 function in a manner similar to colicin M, which uses one copy of its TonB-dependent receptor as both the primary receptor and the translocation pore, or translocation occurs via a second distinct outer membrane translocator protein as for the nuclease E colicins. In the case of the E-type colicins, the primary TonB-dependent receptor, BtuB, does not require an intact TonB box since translocation occurs via the co-receptor or translocator, OmpF. However, for colicin M the TonB-dependent receptor FhuA must carry an intact TonB box for colicin M activity [26,45]. To determine if pyocin SD2 uptake requires the FpvAI receptor to carry an intact TonB box, we tested its ability to kill a PAO1 strain (PAO1Δ*fpvAattP::fpvA*_TBM_) that produces a variant FpvAI carrying an altered (ATAAAAN) and non-functional TonB box [43]. Pyocin SD2 was unable to kill this strain showing that the TonB-FpvAI interaction is essential for pyocin SD2 activity (Figure 4f). Based on the above data, we conclude that pyocin S2/SD2 translocation is mechanistically distinct from that of colicin Ia and the E-type colicins with which they share a highly elongated structure. Instead, since pyocin activity requires a single copy of FpvAI that carries an intact TonB box, it is possible that a single copy of FpvAI acts both as receptor and translocator, in a mechanism similar to colicin M.

### The translocation domain of pyocins S2/SD2 is located at the N-terminus

**Figure 5:**
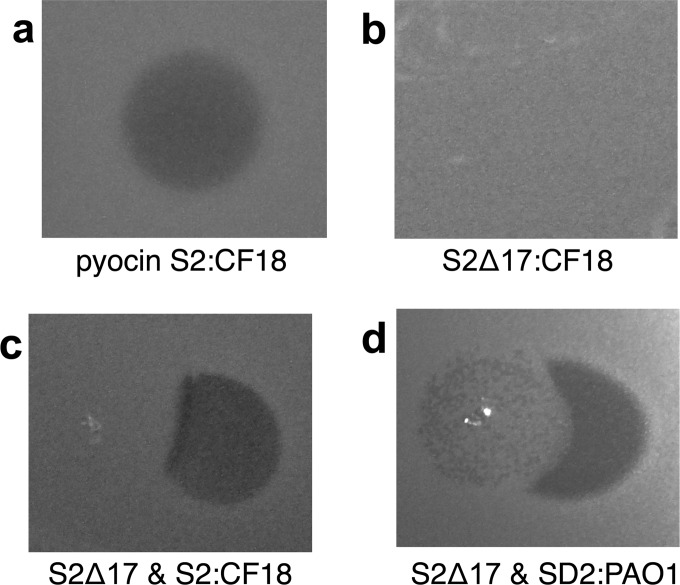
The translocation domain is at the N-terminus of pyocins S2/SD2 Ten microlitres of purified pyocin was spotted on to a growing lawn of cells. Clear zones indicate cell death. (**a**) Inhibition of growth of *P. aeruginosa* CF18 by pyocin S2 (0.1 μg·ml^−1^). (**b**) Pyocin S2Δ17 (750 μg·ml^−1^) is not active against *P. aeruginosa* CF18. (**c**) Inhibition of pyocin S2 killing (0.1 μg·ml^−1^) by pyocin S2Δ17 (750 μg·ml^−1^) against *P. aeruginosa* CF18. (**d**) Inhibition of pyocin SD2 killing (100 μg·ml^−1^) by pyocin S2Δ17 (750 μg·ml^−1^) against *P. aeruginosa* PAO1.

For colicin M both an intact TonB box in the receptor and translocator FhuA and a TonB box located within the intrinsically unstructured translocation domain of the colicin are essential for activity. The first 50 amino acids of pyocins S2/SD2 are proline and glycine rich and thus are likely to have little regular secondary structure, reminiscent of the N-terminal intrinsically unstructured translocation domain of some colicins (Figure 2b). To further probe the mechanism of pyocin S2/SD2 translocation we produced a truncated pyocin S2 variant (pyocin S2Δ17) that lacked 17 amino acids from the N-terminus. This protein was purified via a His_6_-tag on the immunity protein. Formation of the pyocin–immunity protein complex demonstrates proper folding of the cytotoxic domain of this truncated protein. Pyocin S2Δ17 was not active against the pyocin S2-sensitive clinical isolate *P. aeruginosa* CF18 via overlay spot plate assays demonstrating that the first 17 amino acids of pyocin S2 are essential for activity (Figures 5a and 5b). Additionally, the receptor-binding capabilities of this truncated protein remained intact, further demonstrating folding of this truncated protein, as evidenced by overlay spot plate competition assays (Figures 5c and 5d). This shows that although the first 17 amino acids of pyocin S2 are required for killing they are not involved in receptor binding. This suggests a role for the mainly unstructured N-terminal region of pyocins S2/SD2 in recruitment of translocation machinery. If the mechanism of pyocin S2/SD2 is fully analogous to that proposed for colicin M then it would be predicted that a ‘TonB box’ is present within the first 17 amino acids of S2/SD2, although this remains to be confirmed.

These and previous data [16,20] suggest that both the receptor-binding and translocation functionality of pyocins S2/SD2 are housed within the first 216 amino acids. This finding leaves the function of amino acids 217–558 unknown. Therefore, we sought to investigate their role in pyocin activity.

### Pyocins S2/SD2 bind to the CPA of *P. aeruginosa* LPS

**Figure 6:**
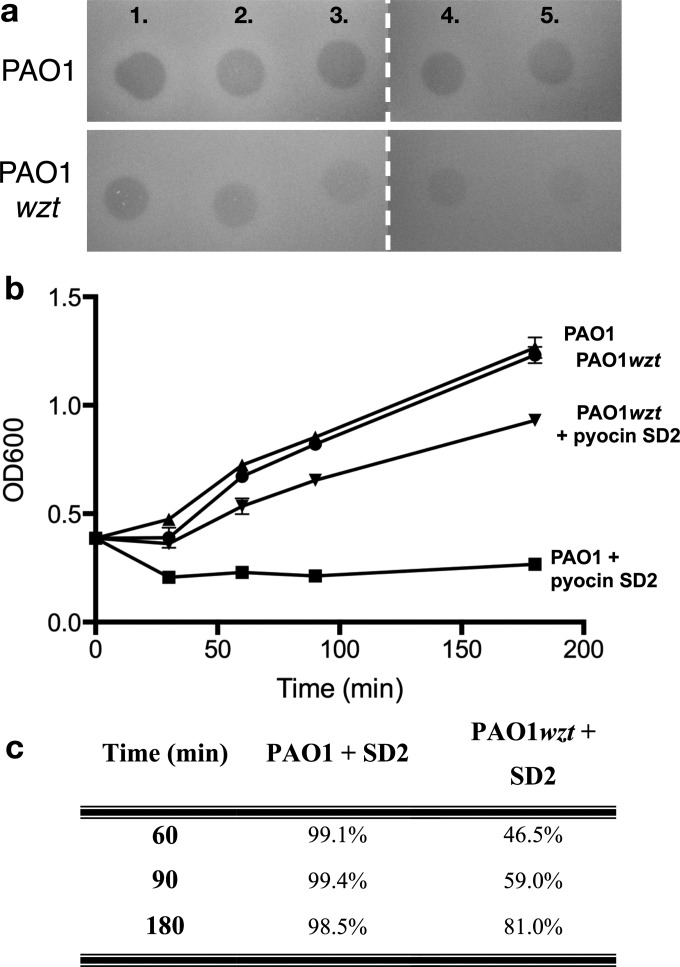
Pyocin SD2 requires the CPA for efficient killing (**a**) Inhibition of growth of *P. aeruginosa* PAO1 and *P. aeruginosa* PAO1*wzt* by pyocin SD2 (1–5: 313 μg·ml^−1^, 2-fold dilutions) as shown by a soft agar overlay spot-test. Ten microlitres of purified pyocin SD2 was spotted on to a growing lawn of cells. Clear zones indicate cell death. (**b**) Representative growth curves for *P. aeruginosa* strains PAO1 and PAO1*wzt* grown in LB broth for 180 min with and without the addition of 3 μM pyocin SD2. *OD*_600_ measured 0, 30, 60, 90 and 180 min after pyocin treatment. Error bars represent the standard error mean between replicate samples (*n*=3). PAO1 (circle), PAO1*wzt* (up triangle), PAO1+pyocin SD2 (square) and PAO1*wzt*+pyocin SD2 (down triangle). (**c**) Percent killing for one of the replicates in (**b**). Ten microlitres of cells (10-fold serial dilutions) were spotted on LB agar plates at 60, 90 and 180 min after pyocin SD2 treatment, incubated at 37°C for 16 h and CFU determined. % killing determined by comparison to untreated controls.

**Figure 7:**
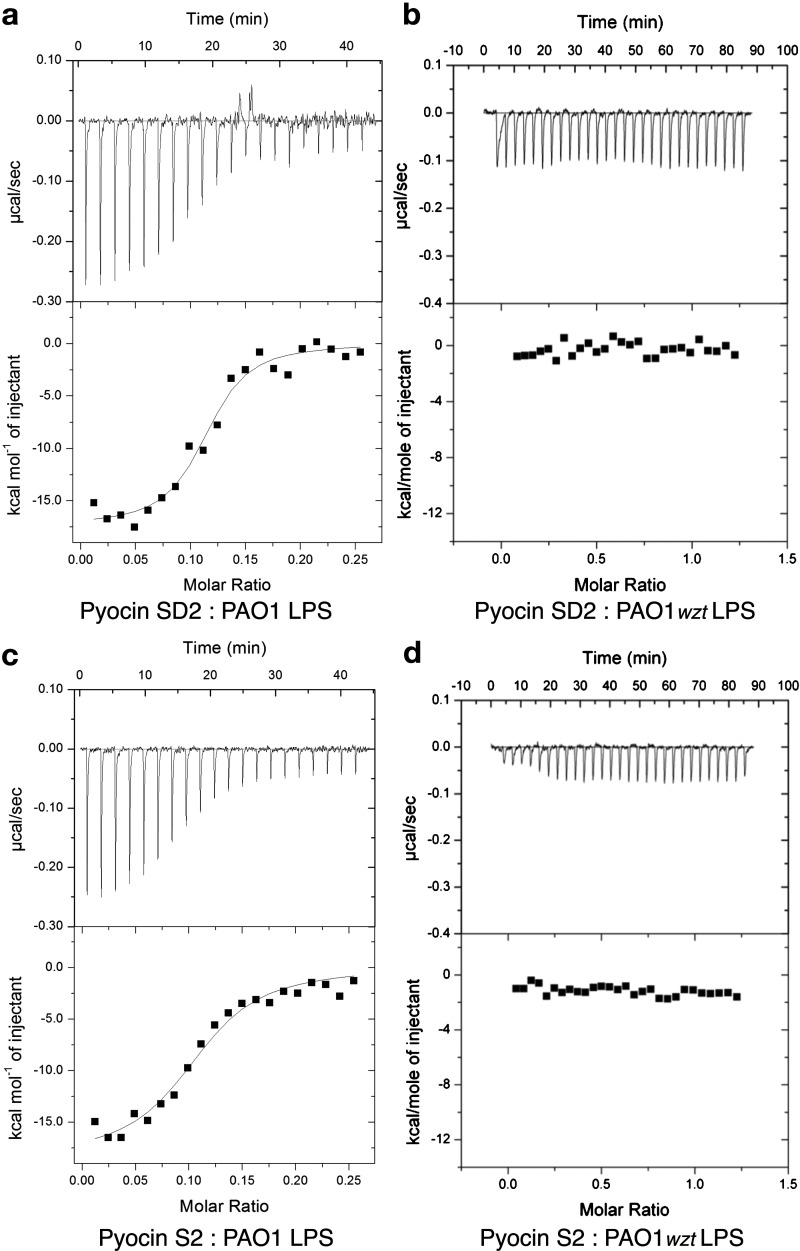
Pyocins SD2 and S2 bind to the CPA of *P. aeruginosa* LPS (**a**) ITC binding isotherm of pyocin SD2 (150 μM) titrated into isolated LPS-derived polysaccharide (3 mg ml^−1^) from wild-type *P. aeruginosa* PAO1. Saturable heats were observed indicative of an interaction. Data were fitted to a single-binding site model that yielded a *K*_d_=0.22±0.02 μM. Reactions were performed in 0.2 M sodium phosphate buffer, pH 7.5 at 30°C using a MicroCal iTC200. (**b**) ITC isotherm of pyocin SD2* (111 μM) titrated into isolated LPS-derived polysaccharide (1 mg·ml^−1^) from PAO1*wzt*. No saturable binding isotherm was observed. (**c**) ITC binding isotherm of pyocin S2 (150 μM) titrated into isolated LPS-derived polysaccharide (3 mg·ml^−1^) from wild-type *P. aeruginosa* PAO1. Data were fitted to a single-binding site model that yielded a *K*_d_=0.62±0.04 μM. Reactions were performed in 0.2 M sodium phosphate buffer, pH 7.5 at 30°C using a MicroCal iTC200. (**d**) ITC isotherm of pyocin S2* (111 μM) titrated into isolated LPS-derived polysaccharide (1 mg·ml^−1^) from PAO1*wzt*. No saturable binding isotherm was observed. Errors reported are the error for the single site binding model fit. *Reactions were performed in 0.2 M sodium phosphate buffer, pH 7.5 at 25°C using a MicroCal VP-ITC.

In recent work we showed that the CPA of *P. aeruginosa* LPS is a cell-surface receptor for pyocin L1, a 32 kDa, globular, lectin-like bacteriocin [21]. To determine if CPA-binding is involved in S-type pyocin activity we assessed the activity of pyocin SD2 against two *P. aeruginosa* PAO1 variants [46], which carry transposon insertions in the genes *wzt* and *wzm*, both in liquid culture and on overlay spot plates (Figures 6a and 6c). The genes *wzt* and *wzm* encode the ATP-binding component and membrane component of a CPA-dedicated ABC transporter. Therefore, these mutants lack the CPA on their cell surface, but still produce the O-specific antigen. The activity of pyocin SD2 against these two mutant strains was reduced when compared with wild type PAO1 suggesting that CPA-binding is involved in pyocin SD2 activity. To determine if pyocin SD2 is able to directly bind the CPA we utilized ITC. ITC binding isotherms for titration of pyocin SD2 into LPS-derived carbohydrates (a mixture of CPA and the O-specific antigen containing polysaccharides) from wild-type PAO1 gave strong saturable exothermic heats of binding with an average *K*_d_=0.58 (±0.36) μM (Figure 7a, Table 2). Conversely, no saturable binding isotherm was obtained when pyocin SD2 was titrated into an equivalent concentration of LPS carbohydrate from PAO1*wzt* (Figure 7b). These data confirm a direct interaction between pyocin SD2 and the CPA of *P. aeruginosa* LPS. Overlay spot plate assays with PAO1*wzt* and high concentrations of pyocin SD2 (300 μM) did not produce central zones of bacterial growth or ‘halos’ within the clear zone of killing, showing a lack of FpvAI saturation in the absence of CPA-binding.

**Table 2:** ITC binding parameters for pyocins titrated into LPS sugars from *P. aeruginosa* PAO1 *Errors in parentheses refer to errors from fit of data from individual experiments to a single-binding site model. ^†^Mean of the two independent experiments (±S.E.M.). ^‡^*N* fixed to 0.10 for fitting (see Materials and methods). ND–not determined.

Pyocin	[Protein] (μM)	[LPS sugars] (mg·ml^−1^)	Δ*H* (±)* (kcal·mol^−1^)	*N* (±)* (sites)	Δ*S* (cal·mol^−1^·deg^−1^)	*K*_d_ (±)* (μM)	Average *K*_d_ (±)^†^ (μM)
SD2	300	3.0	−25.0 (2.4)	0.11 (0.01)	−55	0.93 (0.09)	0.58 (0.36)
SD2	150	3.0	−17.4 (0.7)	0.11 (0.00)	−28	0.22 (0.02)	
S2	75	3.0	−16.6 (0.6)	0.12 (0.00)	−24	0.14 (0.03)	0.38 (0.24)
S2	150	3.0	−18.3 (0.9)	0.11 (0.00)	−33	0.62 (0.04)	
S5	300	3.0	−28.5 (3.9)	0.13 (0.01)	−68	1.94 (0.19)	1.19 (0.75)
S5	150	3.0	−17.9 (0.6)	0.14 (0.00)	−31	0.44 (0.01)	
AP41	100	1.0	ND	–	−	–	–
AP41	150	3.0	ND	–	−	–	
SD1	300	3.0	ND	–	−	–	–
SD1	300	3.0	ND	–	−	–	
SD3	257	3.0	−23.4 (2.8)	0.10 (0.01)	−51	1.84 (0.17)	1.47 (0.37)
SD3	150	3.0	−16.3 (0.9)	0.12 (0.00)	−27	1.10 (0.04)	
S2Δ318	257	3.0	ND	–	−	–	–
S2Δ318	150	3.0	ND	–	−	–	
S2(209)	300	3.0	ND	–	−	–	–
S2(209)	175	1.0	ND	–	−	–	
SD2Δ216	300	3.0	−47.0 (4.0)	0.10^‡^	−134	21.32 (0.55)	22.31 (0.99)
SD2Δ216	300	3.0	−67.8 (5.8)	0.10^‡^	−203	23.29 (0.59)	

As pyocins SD2 and S2 differ only in their cytotoxic domains the above experiment was repeated with pyocin S2 to determine if the CPA-binding determinant of pyocin SD2 is located within this domain. Titration of pyocin S2 into LPS carbohydrates from PAO1 and PAO1*wzt* indicated that the affinity of pyocin S2 for CPA (average *K*_d_=0.38 (±0.24) μM) is comparable to that of pyocin SD2 (Figures 7c and 7d, Table 2). These data show that the CPA-binding motif is located within the N-terminal 558 residues.

**Figure 8:**
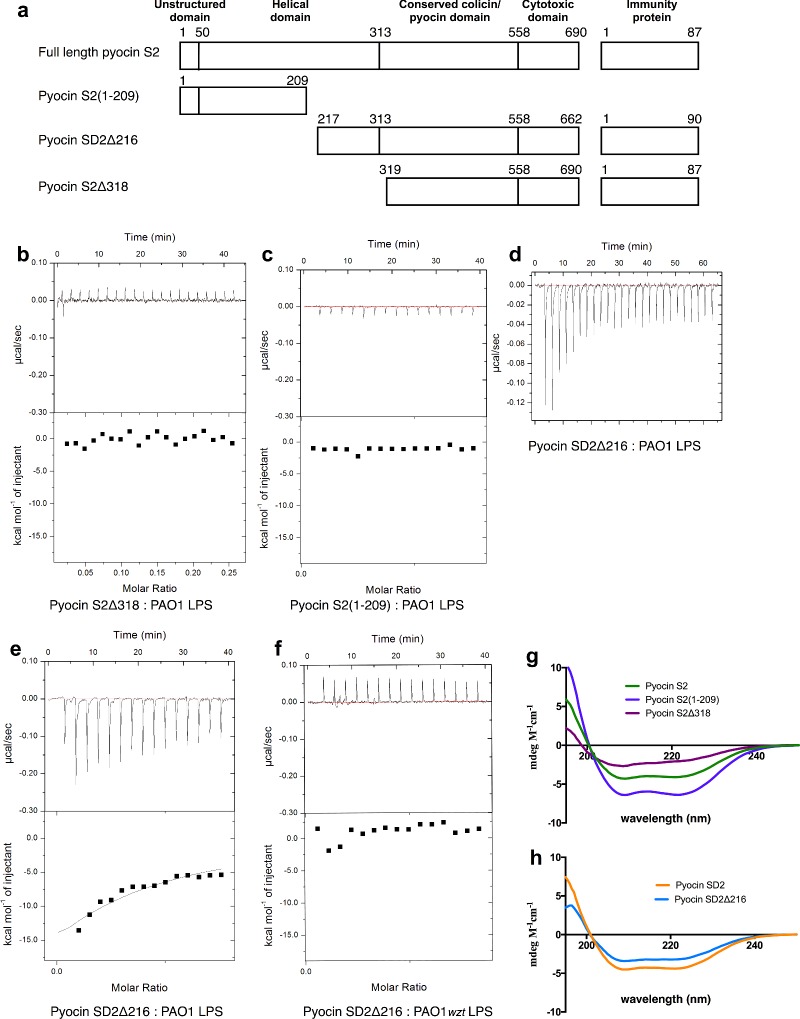
The helical domain of pyocins S2/SD2 is involved in CPA-binding (**a**) Schematic of the protein variants used in this study. (**b**) ITC binding isotherm of pyocin S2Δ318 (150 μM) titrated into isolated LPS-derived polysaccharide (3 mg·ml^−1^) from wild-type *P. aeruginosa* PAO1. No saturable binding isotherm was observed. (**c**) ITC binding isotherm of pyocin S2 (1–209) (300 μM) titrated into isolated LPS-derived polysaccharide (3 mg·ml^−1^) from wild-type *P. aeruginosa* PAO1. No saturable binding isotherm was observed. (**d**) ITC binding isotherm of pyocin SD2Δ216 (888 μM) titrated into isolated LPS-derived polysaccharide (1.5 mg·ml^−1^) from wild-type *P. aeruginosa* PAO1. Saturable heats were observed indicative of a weak interaction. (**e**) ITC binding isotherm of pyocin SD2Δ216 (300 μM) titrated into isolated LPS-derived polysaccharide (3 mg·ml^−1^) from wild-type *P. aeruginosa* PAO1. Heats were observed indicative of a weak interaction. Data were fitted to a single-binding site model, with *N* set to 0.1, that yielded a *K*_d_=23.3±0.59 μM. (**f**) ITC isotherm of pyocin SD2Δ216 (300 μM) titrated into isolated LPS-derived polysaccharide (3 mg·ml^−1^) from PAO1*wzt*. No saturable binding isotherm was observed. Reactions were performed in 0.2 M sodium phosphate buffer, pH 7.5 at 30°C using a MicroCal iTC200. Errors reported are the error of the single site binding model fit. (**g**) Far-UV circular CD spectra were scanned from 250 to 195 nm three times at 25°C for pyocin S2 (green line), pyocin S2(1–209) (blue line) and pyocin S2Δ318 (purple line). All proteins have helical content demonstrating folding. (**h**) CD spectra for pyocin SD2 (orange line) and truncated pyocin SD2Δ216 (light blue line). Both proteins have helical content demonstrating folding. Proteins in 0.2 M sodium phosphate buffer, pH 7.5 at 25°C were diluted to a concentration of 15–150 μg·ml^−1^ and CD spectra were recorded on a J-815 spectropolarimeter (Jasco) in a 0.1 cm quartz cuvette. The final spectra were represented by molar ellipticity, Δ*ε* (mdeg·M^−1^·cm^−1^).

To further dissect the CPA-binding region, three pyocin S2/SD2 truncation constructs were created (Figure 8a). Pyocin S2Δ318, with the first 318 residues (unstructured region and helical region) removed, did not bind to LPS carbohydrates from PAO1, as determined by ITC (Figure 8b, Table 2). This protein variant was purified via a His_6_-tag on the pyocin S2 immunity protein demonstrating protein folding. Far-UV CD also showed this protein to have helical content (characteristic α-helix peaks near 208 nm and 222 nm) (Figure 8g). These data suggest that the CPA-binding motif is located in the first 318 amino acids of pyocins S2/SD2 and not in the CCPD. Pyocin S2(1–209), which consists of the first 209 amino acids only, showed no binding to LPS carbohydrates from PAO1 (Figure 8c). This fragment did retain the ability to bind to the surface of *P. aeruginosa* PAO1 however (Figures 1c and 2d) and far-UV CD analysis showed it to be helical, due to the characteristic α-helix peaks near 208 nm and 222 nm (Figure 8g). This result suggests that amino acids 210–318 are involved in pyocin S2/SD2 CPA-binding. The final construct, pyocin SD2Δ216, weakly interacted with PAO1 LPS-derived carbohydrates (average *K*_d_=22.3 (± 0.99) μM) (Figures 8d and 8e, Table 2). Importantly, this interaction was shown to be specific for CPA as no heats of binding were detected between SD2Δ216 and LPS carbohydrates from PAO1*wzt* (Figure 8f). This protein variant was purified via a His_6_-tag on the pyocin SD2 immunity protein demonstrating protein folding. Far-UV CD analysis of SD2Δ216 gave a secondary structure profile similar to full length pyocin SD2 indicating that the protein is folded (Figure 8h). This mechanism of cell targeting was first reported for pyocin L1, a lectin-like bacteriocin with the conserved sugar-binding motif QxDxNxVxY where x is any amino acid [21]. Although the S-type pyocins do not possess a homologous sugar-binding motif, we show that the major CPA-binding determinant lies within the helical region between amino acids 217–318 of pyocins S2/SD2. However, other regions in the protein may also contribute to the binding of this large substrate or alternatively the binding affinity may have been affected by changes in protein stability caused by the truncation site in the middle of the helical region. These data show that the helical region of pyocins S2/SD2 is involved in both CPA-binding and FpvAI binding.

### CPA-binding by pyocins is a common mechanism of cell targeting

**Figure 9:**
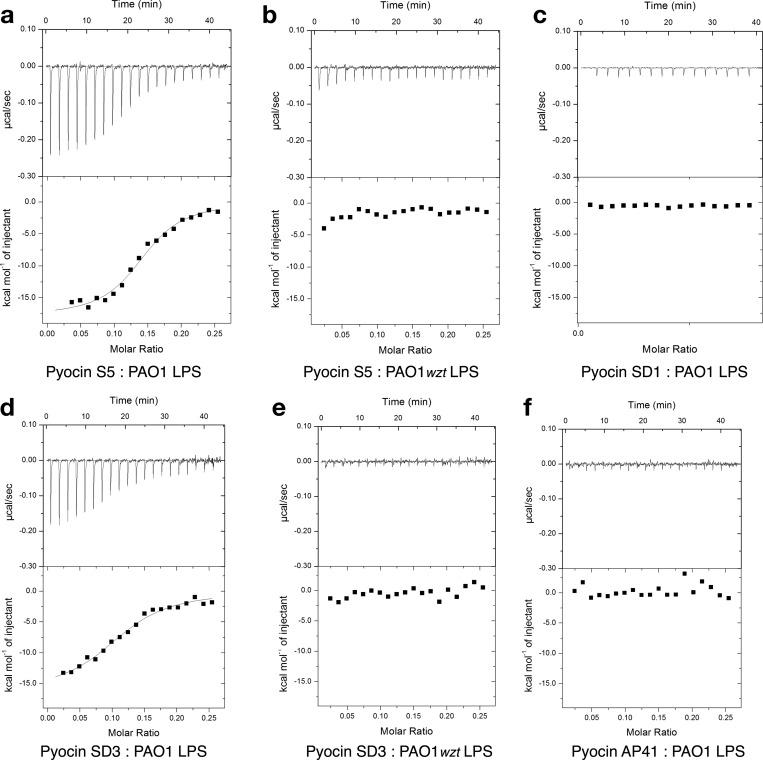
CPA-binding ability of pyocins SD1, SD3, S5 and AP41 (**a**) ITC binding isotherm of pyocin S5 (150 μM) titrated into isolated LPS-derived polysaccharide (3 mg·ml^−1^) from wild-type *P. aeruginosa* PAO1. Strong, saturable heats were observed indicative of a strong interaction. Data were fitted to a single-binding site model that yielded a *K*_d_=0.44±0.01 μM. (**b**) ITC isotherm of pyocin S5 (150 μM) titrated into isolated LPS-derived polysaccharide (3 mg·ml^−1^) from PAO1*wzt*. No saturable binding isotherm was observed. (**c**) ITC isotherm of pyocin SD1 (300 μM) titrated into isolated LPS-derived polysaccharide (3 mg·ml^−1^) from PAO1. No saturable binding isotherm was observed. (**d**) ITC binding isotherm of pyocin SD3 (150 μM) titrated into isolated LPS-derived polysaccharide (3 mg·ml^−1^) from wild-type *P. aeruginosa* PAO1. Saturable heats were observed indicative of an interaction. Data were fitted to a single-binding site model that yielded a *K*_d_=1.1±0.04 μM. (**e**) ITC isotherm of pyocin SD3 (150 μM) titrated into isolated LPS-derived polysaccharide (3 mg·ml^−1^) from PAO1*wzt.* No saturable binding isotherm was observed. (**f**) ITC binding isotherm of pyocin AP41* (150 μM) titrated into isolated LPS-derived polysaccharide (3 mg ml^−1^) from wild-type *P. aeruginosa* PAO1. No saturable binding isotherm was observed. Errors reported are the error of the single site binding model fit. *Reactions were performed in 0.2 M sodium phosphate buffer, pH 7.5 at 25°C using a MicroCal VP-ITC.

Interestingly, amino acids 210–318 of pyocins S2/SD2 share 76% sequence homology to amino acids of 208–300 of pyocin S5. Outside these regions pyocin S5 shares <30% sequence homology with pyocins S2/SD2. As these amino acids house the major CPA-binding determinant of pyocins S2/SD2, the observed homology predicts that pyocin S5 also binds to the CPA. CPA-binding by pyocin S5 was confirmed by ITC using PAO1 LPS-derived carbohydrates [average *K*_d_=1.19 (± 0.75) μM] (Figures 9a and 9b, Table 2). In addition, sequence alignments showed that pyocins S3/SD3 (amino acids 55–150) share 44% sequence identity with amino acids 210–318 of pyocins S2/SD2 and in contrast pyocins S1/SD1 and AP41 share no significant sequence homology with this region of pyocins S2/SD2. Based on these analyses it was predicted that pyocins S3/SD3 would bind to the CPA and pyocins S1/SD1 and AP41 would not. Pyocin SD3 was shown to bind LPS carbohydrates from PAO1 with an average *K*_d_=1.47 (± 0.37) μM and pyocins SD1 and AP41 showed no CPA-binding (Figures 9c–9f, Table 2). This study shows that the strategy of utilizing both the CPA and a protein receptor for cell targeting is common among pyocins. As the majority of *P. aeruginosa* strains produce the CPA [47], targeting of this cell surface motif is an excellent means of concentrating pyocins at the cell surface where they can then search for their receptors/translocators; a mechanism that serves to increase the efficiency of pyocin killing (Figure 6). Importantly in terms of therapeutic applications, CPA is the major surface antigen of *P. aeruginosa* in the cystic fibrosis lung [47–50] and could represent a potential strategy for targeting of anti-pseudomonal therapeutics.

### Pyocin SD2 can afford protection against a lethal *P. aeruginosa* infection *in vivo*

The potency, active uptake and species-specificity of pyocins make them ideal therapeutic candidates for the treatment of *P. aeruginosa* infections. To assess the potential of pyocin SD2 in the treatment of *P. aeruginosa* lung infection *in vivo*, we utilized a murine model. To determine if pyocin SD2 can afford protection against a lethal *P. aeruginosa* infection, mice (*n*=6) were infected intranasally with *P. aeruginosa* PAO1 and treated intranasally 1 h post-infection with pyocin SD2 (25 μl at 3 mg ml^−1^). A second group of mice were treated with pyocin S2 (25 μl at 3 mg·ml^−1^) 1 h post-infection. Pyocin S2-treated mice were included as a control as *P. aeruginosa* PAO1 is a pyocin S2 producer and is therefore resistant to pyocin S2. PBS-treated control mice and pyocin S2-treated mice were culled 6 h post-infection due to the severity of the illness and high levels of bacteria (approximately 10^5^ CFU/lung) were recovered from these mice. Pyocin SD2 treated mice showed no signs of illness and survived to the end-point of the experiment (24 h post-infection). Very low bacterial counts were recovered from the lungs of these mice (5 CFU/lung) and the recovered colonies showed no resistance to pyocin SD2. The efficacy of pyocin SD2 as demonstrated by its ability to afford protection against a lethal *P. aeruginosa* infection, demonstrates the potential of pyocins as potent narrow spectrum therapeutics.

## CONCLUSION

**Figure 10:**
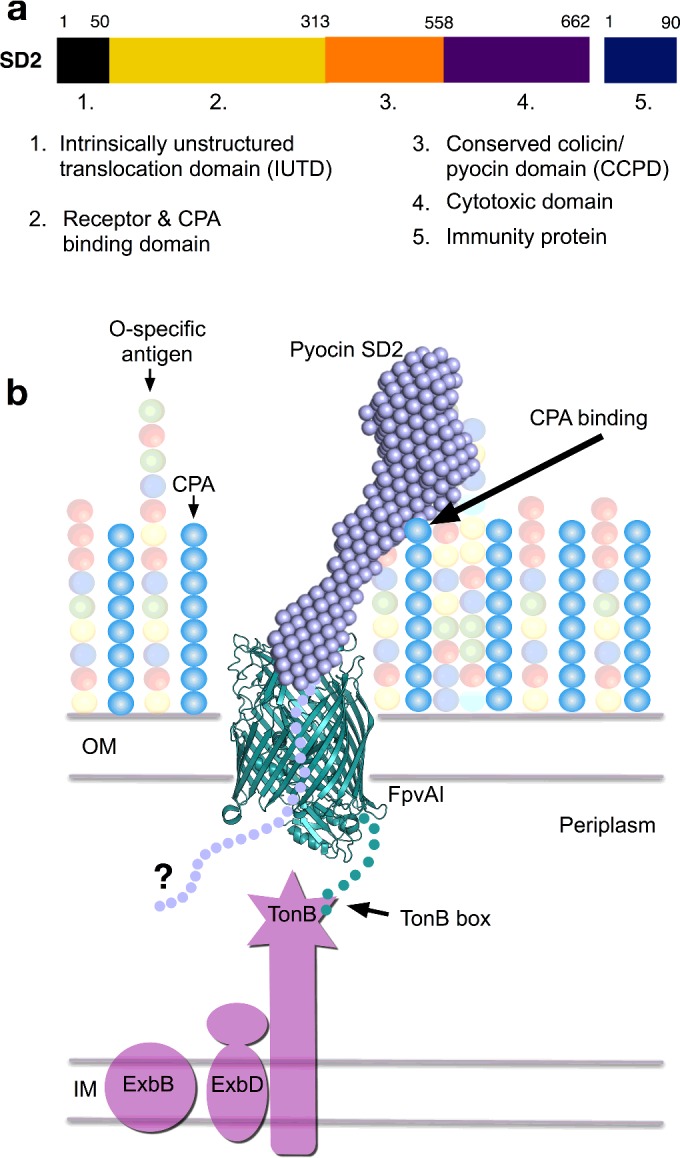
Pyocin binding and translocation model (**a**) Newly proposed domain architecture of pyocin SD2. (**b**) Proposed model of pyocin binding and translocation. Pyocin SD2 binds to the CPA on the cell surface of *P. aeruginosa*, via the receptor and CPA-binding domain, which orientates the N-terminus close to the TonB-dependent outer membrane protein FpvAI. The intrinsically unstructured translocation domain is then positioned to thread through the FpvAI pore and interact with translocation machinery. The interaction of TonB with FpvAI is important for pyocin SD2 translocation across the outer membrane.

Understanding the mechanisms by which biomolecules cross the *P. aeruginosa* outer membrane has the potential to inspire the development of new antibiotics. In this work we propose a model for the cell targeting and translocation of pyocins S2/SD2 across the *P. aeruginosa* outer membrane. In this model, pyocins S2/SD2 are concentrated at the cell surface, via binding to the CPA, from here they can efficiently locate FpvAI in the outer membrane. The requirement for an intact TonB binding box in the FpvAI receptor for pyocin killing indicates that the receptor also plays a role in pyocin translocation although the exact nature of this role and subsequent steps in the translocation process remain poorly understood (Figure 10). Additionally, we show that pyocins are promising therapeutics in their own right. The ability of pyocin SD2 to treat a lethal *P. aeruginosa* lung infection in a murine model shows that pyocin SD2 has excellent antimicrobial activity *in vivo*. The identification of pyocins such as pyocins SD1, SD2 and SD3 also serves to broaden the number of potential pyocin candidates for therapeutic development. In an era where pan-drug resistant *P. aeruginosa* isolates are being more frequently isolated, the development of novel therapeutic approaches to treat *P. aeruginosa* infection, such as those presented in this study, are essential.

## Abbreviations

CFU: colony-forming units
CPA: common polysaccharide antigen
ITC: isothermal titration calorimetry
LPS: lipopolysaccharide
MDR: multi-drug resistance
